# Three-dimensional regional strain analysis in porcine myocardial infarction: a 3T magnetic resonance tagging study

**DOI:** 10.1186/1532-429X-14-85

**Published:** 2012-12-13

**Authors:** Sahar Soleimanifard, Khaled Z Abd-Elmoniem, Tetsuo Sasano, Harsh K Agarwal, M Roselle Abraham, Theodore P Abraham, Jerry L Prince

**Affiliations:** 1 Department of Electrical and Computer Engineering, Johns Hopkins University, 3400 N. Charles Street, Clark Hall 201, Baltimore, MD, 21218, USA; 2 National Institute of Diabetes and Digestive and Kidney Diseases, NIH, Bethesda, MD, USA; 3 Department of Medicine, Division of Cardiology, Johns Hopkins University, Baltimore, MD, USA; 4 Philips Research North America, Briarcliff Manor, NY, USA; 5 Department of Radiology and Radiological Science, Johns Hopkins University, 3400 N. Charles Street, Clark Hall 201, Baltimore, MD, 21218, USA

**Keywords:** Magnetic resonance tagging, Harmonic phase analysis, Three-dimensional regional strain, Myocardial infarction, Diagnostic accuracy

## Abstract

**Background:**

Previous studies of mechanical strain anomalies in myocardial infarction (MI) have been largely limited to analysis of one-dimensional (1D) and two-dimensional (2D) strain parameters. Advances in cardiovascular magnetic resonance (CMR) methods now permit a complete three-dimensional (3D) interrogation of myocardial regional strain. The aim of this study was to investigate the incremental value of CMR-based 3D strain and to test the hypothesis that 3D strain is superior to 1D or 2D strain analysis in the assessment of viability using a porcine model of infarction.

**Methods:**

Infarction was induced surgically in 20 farm pigs. Cine, late gadolinium enhancement, and CMR tagging images were acquired at 11 days before (*baseline*), and 11 days (*early*) and 1 month (*late*) after induction of infarct. Harmonic phase analysis was performed to measure circumferential, longitudinal, and radial strains in myocardial segments, which were defined based on the transmurality of delayed enhancement. Univariate, bivariate, and multivariate logistic regression models of strain parameters were created and analyzed to compare the overall diagnostic accuracy of 3D strain analysis with 1D and 2D analyses in identifying the infarct and its adjacent regions from healthy myocardium.

**Results:**

3D strain differed significantly in infarct, adjacent, and remote segments (p < 0.05) at early and late post-MI. In univariate, bivariate, and multivariate analyses, circumferential, longitudinal, and radial strains were significant factors (p < 0.001) in differentiation of infarct and adjacent segments from baseline values. In identification of adjacent segments, receiver operating characteristic analysis using the 3D strain multivariate model demonstrated a significant improvement (p < 0.01) in overall diagnostic accuracy in comparison with 2D (circumferential and radial) and 1D (circumferential) models (3D: 96%, 2D: 81%, and 1D: 71%). A similar trend was observed in identification of infarct segments.

**Conclusions:**

Cumulative 3D strain information accurately identifies infarcts and their neighboring regions from healthy myocardium. The 3D interrogation of myocardial contractility provides incremental diagnostic accuracy in delineating the dysfunctional and nonviable myocardium in comparison with 1D or 2D quantification of strain. The infarct neighboring regions are the major beneficiaries of the 3D assessment of regional strain.

## Background

Cardiovascular magnetic resonance (CMR) is the gold standard for the assessment of regional myocardial strain, which provides a sensitive and quantitative indicator of myocardial function and viability [1,2]. Several CMR techniques including but not limited to phase-contrast velocity-encoded [3], tagging [4,5], displacement encoding with stimulated echoes [6], and strain encoding [7] imaging are developed to accurately measure one-dimensional (1D) or two-dimensional (2D) strain. These methods have been validated in phantoms, animals, healthy subjects, and patients, and have demonstrated high accuracy and reproducibility in assessment of regional strain [8]. Since 1D and 2D assessment of regional strain requires simplified geometric assumptions on cardiac geometry and deformation, three-dimensional (3D) approaches have more recently been developed and reported to be in agreement with conventional 2D techniques. However, the first 3D approaches required prolonged examinations or sophisticated analysis methods often with extensive manual interactions [8]. Although recent improvements in these techniques, e.g. [9-12], provide better solutions, common practice is still focused on 1D strain (predominantly circumferential strain) [13-16] or 2D strain (predominantly circumferential and radial strains) [17-19], in part due to undocumented benefit of fully 3D strain analysis.

A primary purpose of the present study was to test the hypothesis that quantification of 3D strain is superior to that of 1D (circumferential) and 2D (circumferential and radial) strain quantifications in depiction of regional mechanics and discrimination of viable and nonviable myocardial regions. Accordingly, we studied the contractile mechanics of an *in vivo* porcine model of myocardial infarction (MI). We employed z-encoding harmonic phase (zHARP) [20] and late gadolinium enhancement (LGE) imaging to measure regional strain and myocardial viability, respectively. Univariate, bivariate, and multivariate analyses of regional strain were performed to investigate the diagnostic accuracy of 3D strain analysis compared with 1D and 2D analyses.

## Methods

Animal studies complied with the Institutional Animal Care and Use Committee and conformed to Guide for the Care and Use of Laboratory Animals.

### Experimental preparation

MI was induced in 20 young farm pigs weighing 25 to 35 kg as previously described [21]. In brief, an angioplasty balloon was inserted into the left anterior descending coronary artery (LAD) using an over-the-wire technique under fluoroscopic guidance and inflated to a location just distal to the second diagonal branch of the LAD. Occlusion of the artery was terminated after 150 minutes by deflating the balloon. Post-procedure electrocardiographs showed no prolongation of QRS duration or bundle branch block. All animals were returned to vivarium and received post-operative pain control. Surviving animals, all progressed to heart failure, were monitored daily for the period of the study.

### Image acquisition

CMR images were acquired at 11±6 days (*baseline*) pre-MI, and at 11±4 days (*early*) and 34±8 days (*late*) post-MI. Animals were pre-medicated intramuscularly with droperidol and fentanyl citrate (Innovar 0.1 ml/kg), anesthetized with sodium pentobarbital (20–25 mg/kg, IV), and mechanically ventilated with 1.5% to 2% inhalational Isoflurane for the duration of imaging. Images were obtained using a commercial 3.0 T MR scanner (Achieva, Philips Healthcare, Best, NL) equipped with a six-channel cardiac coil. Animals were placed head first and supine with electrodes for vector electrocardiogram gating [22] on the chest used for respiratory gating. Three types of images were acquired in all animals.

#### Cine

Using multi-slice steady-state free-precession sequence, cine images were obtained during breathholds to study global left ventricular (LV) function. A total of 9 to 12 contiguous short-axis slices were prescribed to cover the LV from base to apex. Image parameters were the following: repetition time (TR) = 3.4 ms, echo time (TE) = 1.69 ms, flip angle (FA) = 40°, field-of-view (FOV) = 280×280mm^2^, matrix size = 256×256, slice thickness = 8 mm (with no gap between slices), and temporal resolution = 20 ms. A total of 39 phases were acquired to cover one cardiac cycle.

#### LGE

Contrast enhanced images were obtained 10 to 15 minutes after intravenous injection of a bolus of 0.2 mmol/kg body weight Gadodiamide (Omniscan GE Healthcare Technologies, Chalfont St. Giles, UK) using a Look-Locker inversion recovery sequence [23] and a 3D free-breathing navigator-gated phase-sensitive inversion recovery (PSIR) gradient echo sequence [24]. Images were acquired at 30 contiguous slices with the same orientation used for the cine images. The PSIR image parameters were: TR = 4.6 ms, TE = 2.2 ms, FA = 15°, FOV = 300×300mm^2^, matrix size = 256×256, and slice thickness = 3 mm (with no gap between slices). The optimal inversion recovery time for optimal suppression of the healthy myocardium was determined by the visual inspection of the Look-Locker images, and it ranged between 200 ms to 250 ms.

#### CMR tagging

zHARP images [20] were acquired during breathholds on the same 9 to 12 short-axis slices used for cine images. This imaging technique, a recently developed and validated tagging technique and an extension to complementary spatial modulation of magnetization [25] and harmonic phase (HARP) analysis [26], enables 3D tracking of short-axis slices using simultaneous in-plane and through-plane displacement encodings and yields 3D strain measures at every pixel in the imaged slices. The images were acquired using a vector electrocardiogram triggered spoiled gradient echo sequence with segmented *k*-space spiral acquisition and spectral-spatial excitation. Image parameters were: TR = 20 ms, TE = 2.5 ms, FA = 15°, FOV = 320×320 mm^2^, matrix size = 256×256, slice thickness = 8 mm (with no gap between slices), spiral interleaves = 12, acquisition window = 12 ms, z-encode frequency = 2π/33 mm [27], and tag spacing = 7 mm. On average, a total of 40 phases were acquired to cover one cardiac cycle. Horizontally and vertically tagged images were acquired in two breathholds per slice.

### Data analysis

**Figure 1:**
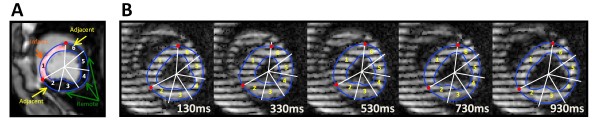
**Division of myocardium into circumferential segments.** (**A**) Epicardial and endocardial borders (blue contours), and borders of transmural MI (red points) are delineated on the LGE image. Myocardial tissue enclosed between MI points is called the infarct segment. The rest of the myocardium is divided into equal segments. Infarct: segment 1; Adjacent: segment 2, and 6; Remote: segment 3, 4, and 5. (**B**) Landmarks delineated on the LGE image are mapped and located on all zHARP cardiac phases by HARP tracking and myocardium is divided into segments accordingly. Five cardiac phases are selected for demonstration.

Cine and LGE images were analyzed using QMass MR software (Medis, Leiden, NL). LV epicardial and endocardial borders, excluding papillary muscles, were traced on end-diastolic and end-systolic cine short-axis images to measure global function. The semi-automated full-width-at-half-maximum method was employed to identify the infarct zone on LGE images [28]. The threshold was adjusted, using n-standard deviations (n < 4) above remote region [28], in a few images where the semi-automated method failed to accurately identify the viable or nonviable regions. Throughout the circumference of the myocardium, infract regions occupying more than 50% of the chord connecting the endocardial contour to the epicardial contour were marked as transmural [29]. LV volume, mid-ventricular wall thickness, stroke volume, ejection fraction, and scar percentage were computed. The cine and LGE images were analyzed prior to the strain analysis, in a blinded manner. For each zHARP slice, the closest LGE slice was identified and mapped to its corresponding mid-diastolic zHARP image using an affine registration [30]. Epicardial and endocardial borders, the anteroseptal right ventricular attachment point (ARVP) to the LV, and the transmural infarct border points previously marked on LGE images were transferred and matched to the mid-diastolic zHARP image. Using HARP tracking [26], those landmarks were identified for all the zHARP cardiac phases. Figure 1 illustrates epicardial and endocardial borders and the transmural infarct border points on an example LGE and five zHARP images at selected cardiac phases.

#### Categorization of myocardial segments

Short-axis slices were categorized into apical, mid, and basal thirds perpendicular to the LV long axis [31] and divided into segments based on viability information provided by the LGE images as shown in Figure 1. The myocardial tissue enclosed circumferentially between the borders of transmural infarct was called the *infarct* segment. The remaining myocardium was divided into segments with equal circumferential width (five segments in basal and mid slices, and three segments in apical slices). The two segments closest to the infarct were called *adjacent* segments and the remaining segments were called *remote* segments. Segments adjacent to an infarct segment in a lower or upper slice were also considered *adjacent* segments. Baseline (pre-MI) images and slices with no evidence of enhancement were divided equally using the ARVP point similar to the 16-segment model [31] (six segments in basal and mid slices, and four segments in apical slices). Segments at baseline were called *healthy* segments.

#### Regional strain endpoints

zHARP images were analyzed with a software developed in-house using MATLAB (MathWorks, Natick, MA). 3D displacement and strain tensors were computed for each pixel of the imaged slices during one cardiac cycle as previously reported [27]. Subsequently, the 3D strain tensors were decomposed into three directional strains (circumferential: *E*_*cc*_, longitudinal: *E*_*ll*_, radial: *E*_*rr*_) based on the gross anatomy of the heart and the orientation of overlying epicardial surface [32]. In addition, 3D strain tensors were decomposed into three principal strains (*E*_*1*_, *E*_*2*_, *E*_*3*_; *E*_*1*_ ≥ *E*_*2*_ ≥ *E*_*3*_) corresponding to the principal directions of deformation. *E*_*1*_ was positive and corresponded to lengthening while *E*_*3*_ was negative and corresponded to shortening. *E*_*2*_, depending on its sign, corresponded to lengthening or shortening. Note that the directional and principal strains are two representations of the 3D strain tensor. Finally, the above-mentioned strain indices were averaged in each segment.

### Statistical analysis

Data was reported as mean ± one standard deviation (SD). One-way analysis of variance (ANOVA) was used to compare LV volume, stroke volume, and ejection fraction at baseline and post-MI time points. Early and late post-MI scar percentages were compared using a paired Student *t*-test. Two-way repeated measures ANOVA was used to compare wall thickness at two cardiac phases (end-systole and end-diastole) and at baseline and post-MI time points. Similarly, two-way repeated measures ANOVA was used to compare strain at the three myocardial segments (infarct, adjacent, and remote) and the two post-MI time points. In addition, ANOVA was used to compare the post-MI strain measures with their baseline values. A Tukey test was used for post hoc analysis if ANOVA was positive.

Post-MI time points were grouped together and univariate (*E*_*cc*_), bivariate (*E*_*cc*_ and *E*_*rr*_), and multivariate (*E*_*cc*_, *E*_*rr*_, and *E*_*ll*_) stepwise logistic regression analyses were performed to evaluate the relation between the strain variables in the healthy segments and the infarct segments, as well as in the adjacent segments. Variables identified with a p-value < 0.05 based on univariate analysis were entered as covariates in the bivariate and multivariate models. A similar multivariate model was created using the 3D principal strain variables (*E*_*1*_, *E*_*2*_, and *E*_*3*_). A receiver operating characteristic (ROC) analysis was used to summarize the overall diagnostic accuracy of the strain models in detecting infarct and adjacent segments from healthy segments. The incremental value of multivariate models was compared with univariate and bivariate models by pair-wise comparison of the c-statistics, equal to the area under the ROC curves using Delong’s method [33]. In all tests, a p-value < 0.05 was considered statistically significant. Statistical analyses were performed using JMP (SAS Institute Inc., Carey, NC) and MedCalc (Mariakerke, Belgium).

## Results

Of 20 animals, 5 died due to incessant ventricular fibrillation (one died intra-operatively during balloon occlusion and four died during the immediate post-operative period). Surviving animals all completed the imaging protocol. Average heart rate was 79 beats/min. Average breathhold durations were 17 s and 18 s for cine and zHARP imaging, respectively.

### Characterization of the infarct model

**Figure 2:**
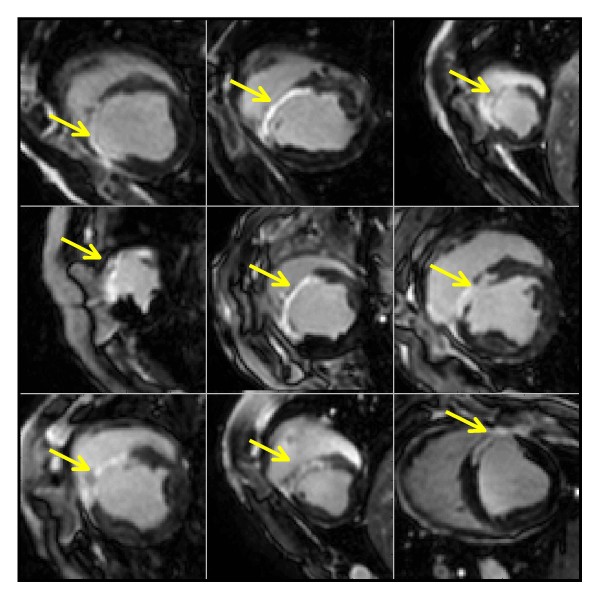
**Example LGE images in nine animals.** Myocardial infarction is mainly located in the anterior wall and anterior septum with transmurality greater than 90% of the wall thickness (yellow arrows). Isolated infarct zones, and slices with sub-endocardial infarct and no presence of transmural infarct were not observed in the entire dataset.

LGE images revealed transmural infarcts in the apical to mid slices and mainly in the anterior wall and anterior septum. Transmurality was greater than 90% of wall thickness and the extension of sub-endocardial infarct was smaller than 25% of the area of adjacent segments in all animals. Isolated infarct zones, and slices with sub-endocardial infarct and no presence of transmural infarct were not observed in the entire dataset. A subset of LGE images is illustrated in Figure 2. Scar size was approximately 16% of LV volume and did not display variation post-MI (early: 16.2±4.9% vs. late: 16.5±4.0%, p = 0.96).

### LV global function and wall thickness

Table 1 summarizes LV global function at baseline and post-MI time points. Increases in stroke volume and LV volume at both end-systole and end-diastole were observed post-MI. Ejection fraction reduced from baseline to early post-MI (43.6±2.8% vs. 26.7±7.6%, p = 0.0105) and then increased at late post-MI (26.7±7.6% vs. 37.5±3.9%, p = 0.0385).

**Table 1:** Global characteristics of left ventricles

	**Baseline**	**Early**	**Late**
**Days after MI**	**--**	**11 ± 4**	**34 ± 8**
**LVEDV (ml)**	**52.5 ± 8.9**	**72.9 ± 14.2**	91.2 ± 27.9*†
**LVESV (ml)**	**29.7 ± 5.9**	**51.0 ± 13.1**	58.6 ± 20.6*
**LVSV (ml)**	**22.8 ± 3.3**	**21.8 ± 9.4**	32.6 ± 8.7*†
**LVEF (%)**	**43.6 ± 2.8**	26.7 ± 7.6*	**37.5 ± 3.9**†
**Scar (%)**	**--**	**16.2 ± 4.9**	**16.5 ± 4.0**

Values are mean±SD. *LVEDV* = left ventricular end-diastolic volume; *LVESV* = left ventricular end-systolic volume; *LVSV* = left ventricular stroke volume; *LVEF* = left ventricular ejection fraction. *p < 0.05 vs. baseline; †p < 0.05 vs. early.

**Figure 3:**
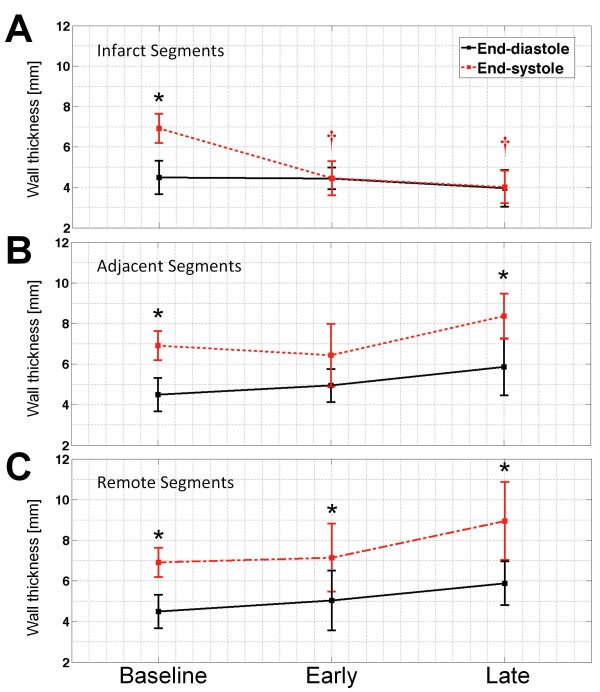
**LV wall thickness.** Mid-ventricular wall thickness is measured in infarct (**A**), adjacent (**B**), remote (**C**) segments at end-systole (red) and end-diastole (black) at baseline and post-MI (*p < 0.05 vs. end-diastole, †p < 0.05 vs. end-systolic baseline.). While no significant wall thickening from end-diastole to end-systole is present in infarct and adjacent segments at early post-MI, the wall thickening in adjacent segments is improved (p < 0.05) at late post-MI. This observation is not present in MI segments.

Mid-ventricular wall thickness values at end-systole and end-diastole are shown in Figure 3. In remote segments, wall thickness at end-systole increased significantly from that at end-diastole at all times (p < 0.001). In addition, a progressive increasing trend in the wall thickness of remote segments was observed from baseline to late post-MI (p = NS) at both end-systole and end-diastole (Figure 3C). In contrast, a significant increase of end-systolic wall thickness from that of end-diastolic, in adjacent segments, was only observed at baseline and late post-MI (p < 0.05). In MI segments, however, there was no post-MI wall thickening from end-systole to end-diastole (p = NS); moreover, end-systolic wall thickness in these segments decreased significantly from baseline values (p < 0.0001).

### 3D Regional strain

**Figure 4:**
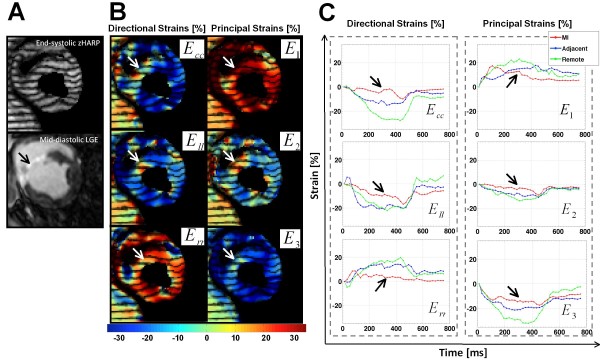
**Example mid-ventricular slice at early post-MI.** (**A**) End-systolic zHARP tagging image (top) with paired mid-diastolic LGE image (bottom). (**B**) End-systolic directional and principal strains overlaid on the zHARP image. Variation of the strain in infarct region from the remote region is consistent with enhancement in LGE image (white arrows). (**C**) Average directional strains (*E*_*cc*_ = circumferential strain, *E*_*ll*_ = longitudinal strain, *E*_*rr*_ = radial strain), and principal strains (*E*_*1*_, *E*_*2*_, *E*_*3*_) are displayed in the infarct, adjacent, and remote segments in one cardiac cycle.

Figure 4A shows an example of an end-systolic zHARP image and its corresponding mid-diastolic LGE image in one animal at early post-MI. Directional and principal strains overlaid on the zHARP image and their temporal profile are shown in Figure 4B and 4C, respectively. Reduced magnitudes were observed in all strain indices in infarct segments versus adjacent and remote segments (Figure 4C, black arrows).

Myocardial 3D regional strain values at baseline and post-MI time points are listed in Table 2. Regional strain values did not demonstrate significant changes in remote segments at both post-MI time points as compared to the baseline healthy segments (p = NS). Directional and principal 3D strain magnitudes reduced significantly in adjacent segments compared to their baseline values as well as that of the remote segments (p < 0.05) at both post-MI time points. A similar finding was observed in infarct segments (p < 0.05). In addition, *E*_*cc*_, *E*_*rr*_, *E*_*2*_, and *E*_*3*_ reduced significantly in infarct segments compared with that in the adjacent segments (p < 0.05). The two-way ANOVA analysis revealed no significant changes between the early and late post-MI time points in any of the six strain measures.

**Table 2:** 3D Regional strain [%] at baseline and post-MI time points

		**Healthy**	**Remote**	**Adjacent**	**Infarct**
**Directional strains**	***E***_***cc***_				
	Baseline	−19.6 ± 3.7			
	Early		−21.0 ± 5.5	−10.8 ± 8.8*†	−3.9 ± 3.7*†‡
	Late		−20.1 ± 5.1	−11.8 ± 4.7*†	−2.5 ± 4.1*†‡
	***E***_***rr***_				
	Baseline	16.4 ± 3.3			
	Early		16.1 ± 4.7	8.8 ± 5.2*†	5.1 ± 5.1*†
	Late		16.8 ± 3.9	7.3 ± 4.8*†	1.6 ± 3.2*†‡
	***E***_***ll***_				
	Baseline	−21.7 ± 6.7			
	Early		−20.3 ± 5.8	−10.9 ± 6.1*†	−8.2 ± 4.2*†
	Late		−19.1 ± 7.4	−10.0 ± 5.1*†	−4.3 ± 5.8*†
**Principal strains**	***E***_***1***_				
	Baseline	24.3 ± 7.7			
	Early		28.1 ± 6.8	19.2 ± 6.1*†	15.5 ± 2.0*†
	Late		26.8 ± 7.0	17.3 ± 5.6*†	12.8 ± 4.3*†
	***E***_***2***_				
	Baseline	−13.1 ± 3.1			
	Early		−12.9 ± 3.5	−7.3 ± 3.9*†	−3.3 ± 2.7*†‡
	Late		−12.0 ± 4.3	−6.8 ± 3.3*†	−3.4 ± 2.8*†‡
	***E***_***3***_				
	Baseline	−25.7 ± 4.6			
	Early		−27.8 ± 4.2	−20.3 ± 4.4*†	−12.8 ± 2.4*†‡
	Late		−26.0 ± 6.1	−20.3 ± 3.3*†	−13.9 ± 2.7*†‡

Values are mean±SD. *p < 0.05 vs. healthy; †p < 0.05 vs. remote; ‡p < 0.05 vs. adjacent.

### Identification of infarct segments by univariate, bivariate, and multivariate models

**Figure 5:**
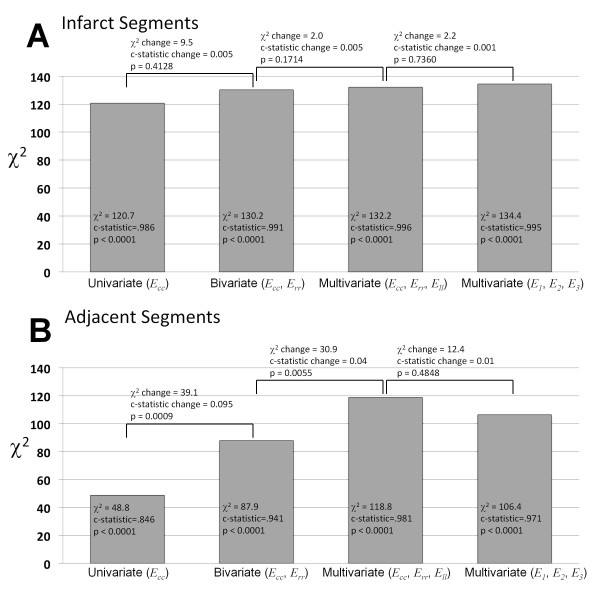
Incremental value of myocardial strain multivariate 3D models over 2D and 1D models in identifying infarct (A) and adjacent (B) segments from healthy segments.

**Figure 6:**
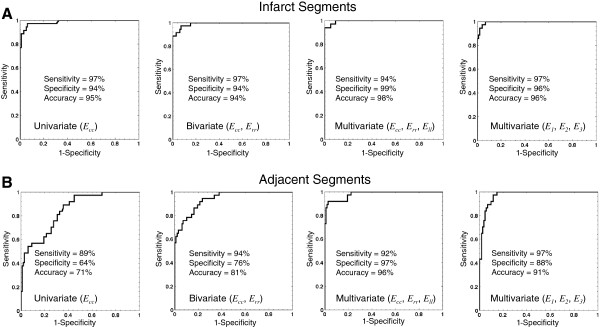
Receiver operating characteristic curves, testing the accuracy of regional strain models in identifying infarct (A) and adjacent (B) segments from healthy segments.

All the three directional strains proved significant factors (p < 0.05) in distinguishing infarct segments from healthy segments by univariate analysis and thus they were entered into the bivariate and multivariate models. The 3D (using *E*_*cc*_, *E*_*rr*_, and *E*_*ll*_) and 2D (using only *E*_*cc*_ and *E*_*rr*_) models demonstrated modest incremental improvement in c-statistic compared with the *E*_*cc*_ univariate model (3D: 0.996, SE, 0.004, CI, 0.964-1.000; 2D, 0.991, SE, 0.006, CI, 0.956-1.000; 1D, 0.986, SE, 0.010, CI, 0.949-0.999, p = NS, Figure 5A). As shown by ROC curves in Figure 6A, *E*_*cc*_ alone provided a powerful metric with a large diagnostic accuracy (95%) in distinguishing infarct segments. The 2D and 3D strain models demonstrated 94% and 98% accuracy, respectively. Similarly, the multivariate model using 3D principal strains, the alternate representation of 3D strain tensor, demonstrated a large c-statistic (0.997, SE, 0.003, CI, 0.964-1.000) in detection of infarct segments with no significant difference from the multivariate model using directional strains (p = NS).

### Identification of adjacent segments by univariate, bivariate and multivariate models

Univariate analysis demonstrated that the three directional strains are independently significant (p < 0.001) in detection of adjacent segments. The incremental value of 2D and 3D models in comparison to 1D *E*_*cc*_ model is shown in Figure 5B. Diagnostic accuracies of all strain models in detecting adjacent segments are smaller than those associated with detection of infarct segments. However, the bivariate model using *E*_*cc*_ and *E*_*rr*_ in which both appeared as significant factors (p < 0.001), demonstrated improved c-statistic (p = 0.0009) compared with 1D univariate analysis using *E*_*cc*_ only (2D: c-statistic, 0.941, SE, 0.020, CI, 0.887-0.974; 1D: c-statistic, 0.846, SE, 0.035, CI, 0.774-0.902). Furthermore, all the strain variables (*E*_*cc*_, *E*_*rr*_, and *E*_*ll*_) were independently significant (p < 0.001) in the 3D analysis and this multivariate model demonstrated even higher diagnostic accuracy (c-statistic, 0.981, SE, 0.010, CI, 0.941-0.997, p = 0.0055). ROC curves for these strain models are shown in Figure 6B. The 1D *E*_*cc*_ model resulted in modest 71% diagnostic accuracy, which was improved to 81% and 96% by 2D and 3D models, respectively. Lastly, the 3D analysis using principal strain variables demonstrated a comparable c-statistic to directional strains in detection of adjacent segments (c-statistic, 0.971, SE, 0.012, CI, 0.926-0.992, p = NS).

## Discussion

Advances in CMR [9-12] allow comprehensive 3D quantification of myocardial contractility without the simplified geometric assumptions commonly associated with 1D or 2D techniques. The clinical adoption of 3D methods however has been hindered in part due to increased imaging time and processing steps required by majority of such methods. Recent developments in CMR imaging and analysis aim to address these issues, and decrease the scanning and processing durations. zHARP, in particular, enables 3D tracking of short-axis slices using simultaneous in-plane and through-plane displacement encodings without affecting the duration of image acquisition. As a result, imaging multiple orientations is eliminated and zHARP yields 3D strain measures at every pixel in the imaged slices without the need for image registration or numerical interpolations [27]. Using zHARP, the present study was designed to assess the value of 3D regional mechanics analysis over the usual in-plane analysis. This incremental value can potentially facilitate the clinical adoption of 3D methods.

In this study, we used a porcine model of MI [34] in which transmural infarction was present in the apical to mid levels. Mean infarct volume was 16% of LV volume with no post-MI variations at 11 days and one month (p = NS). Additionally, classic features of post-MI remodeling was demonstrated in the wall and ventricular chambers (Table 1), accompanied with wall thinning in infarct segments, and wall thickening in remote segments (Figure 3). In the adjacent segments, which contained no transmural infarct and very little sub-endocardial infarct, the wall thickness measurements showed no thickening at early post-MI. Wall thickening in these segments however improved at the late time point. Unlike previous research that reports a decrease in ejection fraction after LV remodeling, an increase in ejection fraction was observed at late post-MI. This observation can be due to mitral valve regurgitation.

For regional strain assessment, the myocardium was divided into segments based on the transmurality and distribution of infarct zone over the circumference of myocardium, characterized by LGE images (Figure 1). Unlike the standard 16-segment model, this method of segmentation eliminated segments with partial transmural infarcts and enabled us to independently study strain patterns in transmural infarcts and the peri-infarct regions that demonstrate different post-MI mechanical behaviors [35]. The transmural infarct consistently spanned the anterior and anterior septal segments, as the infarct was generated following the same procedure and occlusion location in all animals. The size of infarction thereby was largely consistent with a relatively small variation among segment sizes at each cardiac level.

While 3D strain measures in remote segments did not vary significantly from that of the pre-MI healthy segments, the transmural infarct segments and their adjacent segments demonstrated significantly smaller values of 3D strain compared to the remote and healthy regions. Subsequently, univariate, bivariate, and multivariate regression analyses demonstrated that all the directional strain indices (*E*_*cc*_, *E*_*rr*_, and *E*_*ll*_) are significant covariates in identification of adjacent and infarct segments from healthy counterparts. The multivariate model using *E*_*cc*_, *E*_*rr*_, and *E*_*ll*_ resulted in significant improvement (p < 0.01) in c-statistic and diagnostic accuracy (c-statistic = 0.981, accuracy = 96%) compared with the 2D model using *E*_*cc*_ and *E*_*rr*_ (c-statistic = 0.941, accuracy = 81%), and the univariate model using *E*_*cc*_ (c-statistic = 0.846, accuracy = 71%). This finding shows that 3D strain may therefore allow a more accurate and reliable mapping of regional contractility in infarct neighboring regions in comparison with commonly employed 1D or 2D strain measures. However, the 3D strain analysis (c-statistic = 0.996, accuracy = 98%) did not appear significantly superior to simplified single-plane quantification of strain (2D: c-statistic = 0.991, accuracy = 94%, 1D: c-statistic = 0.986, accuracy = 95%) in the presence of a transmural infarct.

The 3D principal strain indices (*E*_*1*_, *E*_*2*_, and *E*_*3*_) corresponded to the three directions of maximal myocardial deformation. *E*_*1*_, corresponding to maximum thickening, was always larger than radial thickening not only in the infarct segments but also in the remote, and adjacent segments (Table 2). Similarly, the maximum shortening values, *E*_*3*_, were larger (in absolute value) than the corresponding circumferential strain values in all segments at all time points. These principal strain indices demonstrated a precise assessment of regional function and did not illustrate inferior diagnostic performance in comparison with the more commonly used directional strain indices (Figure 6). The decomposition of principal strain indices from 3D strain tensor, unlike directional strain indices, does not depend on the gross geometry of LV and the orientation of imaged slice. This universal aspect of principal strains and their insensitivity to image planning may result in more accurate and robust strain values in comparison to *E*_*cc*_, *E*_*rr*_, and *E*_*ll*_. However, this speculation requires a more careful study to be confirmed. The direction of principal strain may also provide a regionally varying index of myocardial deformation. However, the extraction of angles and averaging those angles within each segment must be performed under special care especially in regions with infarct, which may lack a dominant direction of deformation. Definitive conclusion warrants further investigation and analysis, which is beyond the scope of the present work.

CMR-based 3D strain may therefore allow accurate and reliable mapping of regional contractility and assessment of viability. 3D analysis may also have implications beyond analysis of myocardial infarction and it may help in assessing patients with ischemic or non-ischemic cardiomyopathy [36]. The present study may help lay the road for a larger follow up study, with a longer post-MI time period and a wider spectrum of MI size, to investigate if an early strain signature can predict post-MI remodeling and determine the salvageable myocardium.

### Limitations

The MI model used in this study was approximately 16% of the LV volume and restricted to the apex and septum. Furthermore, the MI was mainly transmural and the extent of sub-endocardial infarct was small (<25% area of adjacent segments). Infarctions in clinical practice that are not revascularized in time usually present a wider spectrum of MI size and distribution with the potential for larger sub-endocardial infarcts. The latter condition would present the ideal condition for 3D regional function analysis. The segmentation method for the baseline images and the post-MI images was different, which prevented direct comparison. Also, we did not perform pathologic validation of our model. However, this porcine model of MI has been characterized using high resolution *ex vivo* CMR [37] and has been reported to exhibit all the morphologic and functional remodeling features of clinical infarctions [34]. There is also extensive published literature on the spatial correlation of infarct and its neighboring regions with delayed enhancement. Similarly, we did not perform a dobutamine challenge to assess the viable segments. This concept also has been validated repeatedly with regards to delayed enhancement. Lastly, we did not assess mitral valve regurgitation, which could potentially explain the increase in ejection fraction at the late post-MI time point.

## Conclusions

Multivariate 3D strain analysis using CMR accurately differentiates infarct from normal myocardium and correlates with delayed enhancement. Furthermore, cumulative 3D strain is superior to 1D and 2D strain analyses for identification of infarct neighboring regions. Further evaluation of this technique in human subjects will help determine the clinical value of 3D regional strain quantification.

## Competing interests

Jerry L. Prince is a co-founder of Diagnosoft Inc., a company that licensed the HARP technology. The terms of this arrangement are managed by the Johns Hopkins University in accordance with its conflict of interest policies. Other authors have declared no competing interests.

## Authors’ contributions

SS: substantial contributions to design of study, analysis and interpretation of data, and drafted the manuscript. KZA: substantial contributions to design of study, acquisition and interpretation of data, and revision of manuscript. TS: substantial contributions to acquisition of data. HA: substantial contributions to interpretation of data. MRA: substantial contributions to design of study, interpretation of data, and revision of manuscript. TPA: substantial contributions to design of study, interpretation of data, and revision of manuscript. JLP: substantial contributions to design of study, interpretation of data, and revision of manuscript. All authors read and approved the final manuscript.
